# Individualized perioperative nutritional management in orthognathic surgery: an evidence-graded, integrated framework linking upper-airway change, feeding function, and psychological status

**DOI:** 10.3389/fnut.2026.1944347

**Published:** 2026-09-08

**Authors:** Cuicui Ge, Mingjun Zhang, Dongyue Yao

**Affiliations:** 1 Department of Emergency and Mucosal Diseases, Hospital of Stomatology, Jilin University, Changchun, China; 2 Department of Craniomaxillofacial Plastic and Cosmetic Center, Hospital of Stomatology, Jilin University, Changchun, China

**Keywords:** body dysmorphic disorder, individualized nutritional management, masticatory function, obstructive sleep apnea, orthognathic surgery, perioperative nutrition, upper airway

## Abstract

Orthognathic surgery corrects dentofacial deformity and increasingly addresses upper-airway obstruction, but postoperative dietary restriction may cause inadequate intake, weight loss, and lean-mass depletion. Nutritional care remains underexamined and rarely treated as a multidimensional problem. This mini-review synthesizes evidence across three interacting determinants of postoperative nutrition: upper-airway morphological change, temporary impairment of mastication and swallowing, and perioperative psychological status. We grade each domain by its proximity to measured nutritional outcomes because the evidence is markedly asymmetric. Only feeding function has direct longitudinal support: intake, weight, muscle mass, and serum markers track masticatory recovery, with intake lowest in week one, weight recovering by about 4 months, and muscle mass remaining incompletely restored at 6 months. Evidence for airway and psychological pathways remains indirect. Mandibular setback reduces airway volume and may increase obstructive sleep apnea risk, whereas advancement enlarges the airway; both may alter swallowing mechanics. Anxiety, depression, and body dysmorphic disorder are also common, but no study has related airway change or psychological status to intake, weight, or body composition in this population. Effects cannot be extrapolated from general populations, where sleep restriction may increase energy intake, and depression affects appetite bidirectionally. We therefore present an explicitly hypothesis-generating, risk-stratified framework. Its feeding-function arm can inform practice; its airway and psychological arms support low-risk precautions and targeted research. We outline minimum study designs needed to convert inferred links into measured ones and identify priorities for individualized nutritional management.

## 1 Introduction

Orthognathic surgery corrects dentofacial deformity and improves occlusal function and facial esthetics by osteotomizing and repositioning the maxilla and mandible. Advances in computer-aided design and patient-specific fixation have expanded indications from purely occlusal correction to the management of upper-airway obstruction (1). Perioperative care nonetheless remains challenging, and nutritional management is a long-underestimated but critical component.

After surgery, patients typically face weeks to months of dietary restriction. Postoperative maxillomandibular fixation, soft-tissue swelling, pain, and limited mouth opening impede normal oral intake and can lead to inadequate nutrition and marked weight loss (2, 3). A systematic review reported near-universal reductions in postoperative weight and body mass index (BMI), with mean maximal weight loss reaching 4.1 kg and greater decline at higher preoperative BMI (4, 5). Such changes impair wound healing, may prolong hospitalization, and increase complication risk (2). Critically, perioperative nutrition is not an isolated problem: postoperative airway remodeling (6, 7), transient masticatory and swallowing dysfunction (8, 9), and shifting psychological status (10–12) are interwoven and jointly determine a patient’s capacity and willingness to eat.

**Figure 1:**
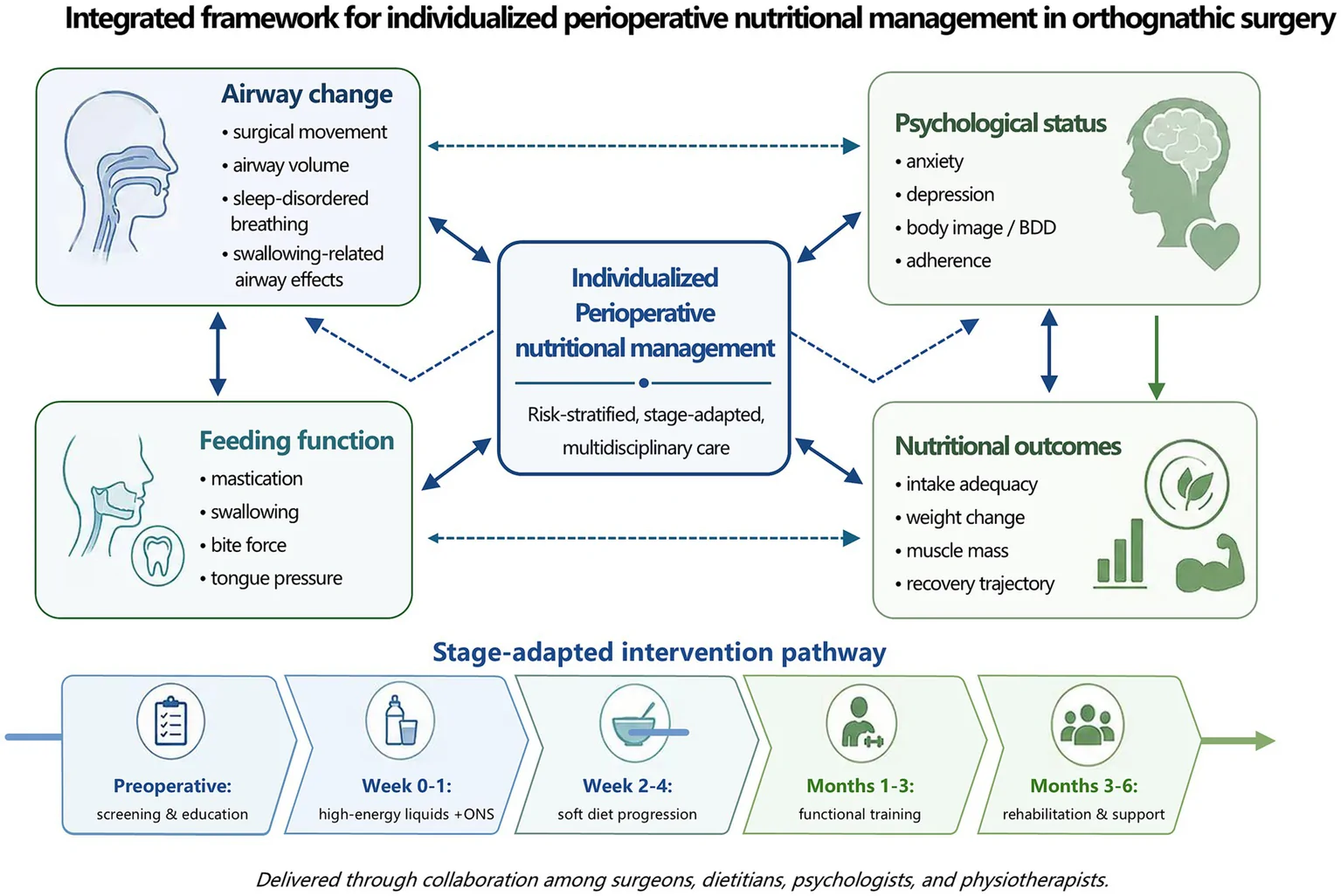
Integrated framework for individualized perioperative nutritional management in orthognathic surgery. The framework illustrates how surgically induced upper-airway changes, postoperative feeding-function impairment, and perioperative psychological status are proposed to interact to shape nutritional outcomes after orthognathic surgery. Link strength is now shown explicitly: Solid arrows denote links supported by measured nutritional endpoints in orthognathic patients, and dashed arrows denote links that are inferred from intermediate endpoints or mechanisms and remain untested. Only the feeding-function-to-nutrition arm is solid. Airway-related changes may influence sleep-disordered breathing and swallowing safety, while impaired mastication, swallowing, bite force, and tongue pressure directly restrict oral intake. Psychological factors, including anxiety, depression, body-image disturbance, and adherence, are hypothesized to further modify dietary behavior and recovery, with a reciprocal arrow from prolonged dietary restriction to psychological distress, the direction for which qualitative evidence exists. Individualized nutritional management should therefore be risk-stratified, stage-adapted, and delivered through multidisciplinary collaboration. The lower timeline summarizes a staged intervention pathway from preoperative screening and education to early high-energy liquid nutrition, soft-diet progression, functional training, and longer-term rehabilitation and psychological support.

Existing literature largely examines these issues one dimension at a time and rarely integrates airway, feeding function, and psychological factors. The three domains are also not equally well evidenced: only for feeding function have nutritional endpoints themselves been measured, whereas elsewhere the published chain terminates at an intermediate outcome such as pharyngeal volume, apnea-hypopnea index or a symptom score, and the final step to intake, weight or body composition is supplied by reasoning rather than data. Treating this asymmetry as a finding rather than concealing it is a central aim. This mini-review appraises how the three domains influence nutritional management, how strong the evidence for each link actually is, highlights controversies and gaps, and proposes an evidence-graded integrated framework for individualized nutritional management (Figure 1).

## 2 Literature search and evidence appraisal

This narrative mini-review synthesizes evidence on the interplay among airway change, feeding function, psychological status, and nutritional management in orthognathic surgery. PubMed/MEDLINE, Embase, Web of Science, and the Cochrane Library were searched from database inception to 30 June 2026, combining population terms (orthognathic surgery, dentofacial deformity, sagittal split osteotomy, Le Fort I, maxillomandibular advancement or setback) with domain terms for nutrition, upper airway and obstructive sleep apnea, feeding function, and psychological status; reference lists were screened manually. English-language human studies reporting a nutritional, airway, feeding-function or psychological outcome after orthognathic surgery were eligible, plus studies in other populations cited explicitly to test the direction of an inferred link; case reports, conference abstracts, and syndromic, trauma or oncological cohorts were excluded. Search strings and full eligibility criteria are given in Supplementary Tables S1, S2.

Because the aim was integrative synthesis rather than quantitative pooling, evidence was appraised narratively, with emphasis on the direction, magnitude, and temporal pattern of effects. The methodological quality of every study supporting a domain-to-nutrition link was nevertheless assessed formally with design-appropriate instruments: the Newcastle-Ottawa Scale for observational studies (13), RoB 2 for randomized trials (14), ROBINS-I for non-randomized intervention studies (15), AMSTAR 2 for systematic reviews (16), and the CASP checklist for qualitative studies (17); item-level ratings appear in Supplementary Tables S3–S7. Each link was additionally graded by its proximity to a nutritional endpoint: direct, where studies in orthognathic patients measured intake, weight, body composition, or nutrition-related biochemistry as a function of the domain variable; indirect, where only an intermediate outcome was measured; and inferred, where the link rests on mechanism or extrapolation from other populations. Proximity and quality are independent axes—several methodologically sound studies support only indirect links—so both are reported per link in Supplementary Table S8; the proximity grades are used throughout and summarized in Table 1.

**Table 1:** Multidimensional nutritional-risk domains, graded strength of the link to measured nutritional outcomes, and stage-adapted management implications in orthognathic surgery.

Perioperative domain	Nutrition-related mechanism	Evidence grade for the link to nutritional outcomes	Key evidence and time course	Implications for individualized nutritional management
Upper-airway remodeling and sleep-disordered breathing	Procedure-dependent changes in pharyngeal volume, minimum cross-sectional area, tongue position, and hyoid position are proposed to influence breathing during sleep and swallowing safety, thereby potentially modifying feeding tolerance and intake.	Indirect. Airway volume, minimum cross-sectional area, apnea-hypopnea index and swallowing physiology are measured, but no study reports intake, weight or body composition as a function of these variables. The direction of any sleep-mediated effect is unresolved, since sleep restriction increases energy intake in healthy adults (23).	Mandibular setback may reduce airway volume and raise OSA risk, whereas advancement procedures generally enlarge the airway. Early edema can aggravate narrowing during the period of greatest dietary restriction; partial recovery is usually assessed over 3–6 months (6, 18–22, 24, 25).	Use preoperative airway assessment, especially in large setback or high-risk patients. In the early postoperative stage, avoid feeding positions or textures that may worsen obstruction or aspiration risk; coordinate nutritional care with airway and sleep-symptom monitoring. Precautionary rather than nutritionally proven.
Masticatory and swallowing dysfunction	Limited mouth opening, pain, occlusal instability, reduced bite force, altered tongue pressure, and impaired chewing efficiency directly restrict oral intake and food-texture tolerance.	Direct. Energy, fat and fibre intake, body weight and BMI have been measured longitudinally in orthognathic patients and track the timing of functional recovery.	Energy and fat intake decline in the first postoperative week, fiber intake may remain low for 1–2 weeks, and functional recovery often extends toward 3 months. Evidence for chewing-training protocols remains mixed (3, 4, 8, 9, 26, 28).	Adopt staged texture progression: high-energy full liquids in week 1, gradual transition to soft diet during weeks 2–4, and functional chewing/swallowing rehabilitation when clinically appropriate. Adjust texture, meal frequency, and protein density to functional recovery.
Dietary restriction, weight loss, and body-composition depletion	Reduced intake and prolonged texture restriction can cause weight loss, lean-mass depletion, and early changes in hemoglobin and albumin; weight alone may underestimate nutritional burden.	Direct. Weight, BMI, bioimpedance-derived muscle mass, hemoglobin and albumin have been measured serially, and oral nutritional supplementation has been tested in a randomized trial.	Weight loss and BMI reduction are common, with greatest loss often early after surgery. Muscle mass may fall substantially in the 1 month and remain incompletely recovered at 6 months. ONS may attenuate early weight loss, but sustained benefits are uncertain (2–5, 27, 29, 30).	Prioritize high-protein, energy-dense liquid/soft diets and consider ONS for high-risk or poorly tolerant patients. Monitor body weight, intake, muscle mass where feasible, and laboratory markers rather than relying only on BMI.
Psychological status, body image, and adherence	Anxiety, depression, BDD symptoms, pain catastrophizing, insomnia, and low resilience may reduce appetite, increase eating avoidance, and weaken adherence to diet and rehabilitation regimens.	Inferred. Symptom prevalence and trajectories are well measured, but no orthognathic study reports a nutritional endpoint as a function of psychological status. Appetite change in depression is bidirectional (34). The one direct co-observation runs the reverse way, from dietary restriction to distress (31).	Preoperative anxiety and BDD symptoms are common; early postoperative anxiety and depression are frequent and may evolve over 3–6 months. Qualitative evidence shows dietary restriction can create social isolation and psychological distress (10–12, 31, 33, 35–41).	Include psychological screening in nutrition-risk stratification. Provide anticipatory dietary education, expectation management, adherence support, and referral pathways for high-anxiety, BDD-positive, or low-resilience patients. Justified by psychological benefit and low burden, not by proven nutritional gain.
Integrated risk stratification and long-term follow-up	Airway change, feeding-function impairment, and psychological status are hypothesized to interact; nutritional recovery is therefore modeled as a multidimensional trajectory rather than a single postoperative diet problem.	Untested. No study measures cross-domain mediation. The integrated model is hypothesis-generating and should be evaluated by cohorts that record airway, feeding, psychological and nutritional variables concurrently.	Protocols are heterogeneous, and studies testing cross-domain mechanisms or integrated interventions are scarce. Weight recovery may take >4 months, while muscle mass and psychological symptoms may require longer monitoring (4, 27, 33, 43, 44).	Use a multidisciplinary pathway involving maxillofacial surgeons, dietitians, psychologists, and rehabilitation professionals. Digital tools may support intake records, weight tracking, and PROMs, but require validation.

BDD, body dysmorphic disorder; BMI, body mass index; ONS, oral nutritional supplements; OSA, obstructive sleep apnea; PROMs, patient-reported outcome measures. Evidence grades: direct, a nutritional endpoint (intake, weight, body composition, or nutrition-related biochemistry) was measured in orthognathic patients as a function of the domain variable; indirect, only an intermediate endpoint was measured; inferred, the link rests on mechanism or on extrapolation from other populations.

## 3 Upper-airway change and nutritional management: an indirect link

The upper-airway effects of orthognathic surgery are well documented and differ in direction and magnitude by procedure. Evidence here is graded indirect throughout: airway change is precisely quantified, but none of the studies reviewed recorded a nutritional endpoint. A systematic review and meta-analysis found that setback, alone or combined with maxillary advancement, reduced total upper-airway volume, whereas isolated mandibular and bimaxillary advancement increased it; beyond 3 months, volume fell by approximately 6,042 mm^3^ after setback and rose by approximately 7,967 mm^3^ after bimaxillary advancement, although only the former reached significance (6). A three-dimensional analysis reported an immediate volume increase of about 33.4%, with bimaxillary advancement and counterclockwise mandibular rotation contributing most (18).

Two pathways have been proposed to link airway change to nutrition; both rest on intermediate rather than nutritional endpoints. First, in skeletal Class III patients undergoing setback, reduced volume may raise the risk of obstructive sleep apnea (OSA): although most patients do not develop clinically significant OSA, meta-analysis shows upper-airway volume and minimum cross-sectional area remain significantly below baseline at 6 months (19, 20). Reported postoperative OSA incidence varies with procedure and setback magnitude: 19.2% after Le Fort I plus bilateral sagittal split setback, 8.57% after isolated sagittal split setback, and only 0.7% when maxillary advancement accompanied setback (21). The step from disturbed sleep to impaired nutrition is inferred, not observed: studies assessing sleep in these patients related it to satisfaction and quality of life, not to intake or weight (22), and the general sleep literature does not license extrapolation, since experimental sleep restriction in healthy adults increases energy intake by roughly 250 kcal/day (23). Whether postoperative sleep fragmentation reduces intake, or has little net effect once texture restriction is accounted for, is unknown; even the direction remains open.

Second, airway change is closely linked to swallowing. Postoperative repositioning of the hyoid bone and tongue may affect pharyngeal closure during deglutition. An electromyographic study after mandibular setback found a shortened oral preparatory phase and disappearance of piecemeal deglutition in some patients (24)—compensatory changes, measured as swallowing physiology rather than bolus volume, meal duration or energy delivered. We could identify no orthognathic study reporting nutrient intake, weight change or body composition as a function of measured airway volume, minimum cross-sectional area, apnea-hypopnea index or swallowing kinematics. The airway-to-nutrition link is at present a plausible mechanism without confirmatory data.

Airway effects are also time-dependent: during the first postoperative month, edema can aggravate narrowing precisely when dietary restriction is greatest (2), and volume partially recovers by 3–6 months but rarely returns to baseline (19, 25). This temporal overlap yields a falsifiable prediction: within a setback cohort, patients in the highest tertile of airway-volume reduction should show lower early energy intake and slower weight recovery than those in the lowest, after adjustment for extent of surgery, maxillomandibular fixation and analgesia. Testing it requires only that existing airway cohorts add an intake record and serial body-composition measurement. Until such data exist, stage-adapted precautions such as avoiding feeding positions and textures that could worsen obstruction or aspiration rest on swallowing-safety reasoning, not demonstrated nutritional benefit.

## 4 Feeding-function change and nutritional intervention: the direct link

Changes in feeding function are the most direct determinant of postoperative nutritional status and the only domain in which nutritional endpoints have actually been measured: studies report intake, weight, bioimpedance-derived muscle mass, hemoglobin and albumin against the timing of functional recovery. Reduced masticatory efficiency, limited mouth opening, oral pain, and occlusal instability together impede early oral intake. A prospective longitudinal study found significant declines in energy and fat intake during the first postoperative week, with fiber intake reaching its nadir in weeks one to two and recovering to baseline only by 3 months (26). Body weight followed the same pattern, with greatest loss at week one and most patients regaining preoperative weight only by 4 months (3, 4). Bimaxillary procedures produced greater weight loss than single-jaw surgery, and men lost more than women (3, 27).

The trajectory of masticatory recovery guides intervention design. Electromyography shows persistent masseter-temporalis imbalance at 3 months, indicating incomplete normalization of chewing patterns (8); chewing-gum training improves this balance (8), yet a randomized trial found that adding a chewing exerciser to conventional physiotherapy conferred no additional benefit, implying that protocol and intensity require optimization (9). Tongue pressure is also relevant: in mandibular prognathism, swallowing generates pressure first at the peripheral palate with delayed offset timing, suggesting that swallowing retraining should address tongue-pressure recovery (28).

Nutritional intervention centers on texture progression and supplementation, but protocols vary widely. A survey found the most common regimen to be full liquids for 2–4 weeks transitioning to a soft diet for 2–6 weeks, yet identified seven distinct schemes, reflecting the absence of unified guidelines (4). The benefit of oral nutritional supplements (ONS) with counseling remains contested: a randomized trial showed less weight loss in the ONS group at weeks two and four, but no difference in nutritional parameters or oral-health-related quality of life by week twelve (29).

Nutritional change extends beyond weight and BMI to body composition. Bioelectrical impedance showed mean muscle-mass loss of about 1.79 kg in the 1 month, with incomplete recovery by 6 months (27). Hemoglobin and serum albumin fall early, and albumin may remain below baseline at 6 months (2, 30). Because energy repletion alone may not reverse muscle loss, optimizing protein quantity and quality should be a central goal.

Clinically, management should be staged: week one warrants high-energy, high-protein full liquids with ONS where needed; weeks two to four allow gradual transition to a soft diet, avoiding foods requiring vigorous chewing; and 3 months is a key window for masticatory recovery, when chewing training may be most effective. Importantly, dietary restriction also imposes a psychosocial burden: a qualitative study found that patients, often under-informed, fall into a negative spiral of social isolation and distress (31)—the one setting in which nutrition and psychology have been observed together, with the direction running from restriction to distress rather than the reverse. Nutritional intervention should therefore incorporate education, psychological support, and social strategies alongside macronutrient provision.

## 5 Psychological factors and nutritional management: an inferred and probably bidirectional link

Perioperative psychological status is complex, and its influence on nutrition remains underappreciated and, so far, unmeasured. Preoperative anxiety is among the commonest problems: a cross-sectional study reported high anxiety in 25.8–32.7% of preoperative patients and clinically significant body dysmorphic disorder (BDD) symptoms in 22.9% (12). A multicenter study found social support, resilience, and satisfaction with clinical-team information negatively associated with preoperative anxiety, and avoidant coping positively associated (11); qualitative work showed anxiety to be multifactorial, encompassing fear of surgery, uncertainty, need for control, and trust-building (32).

Postoperative depression and anxiety are also substantial. A prospective study reported depression in 32% and anxiety in 72.8% of patients within five postoperative days, with preoperative pain catastrophizing predicting depression, and preoperative anxiety, moderate-to-severe pain, nausea and vomiting, and insomnia predicting anxiety (10). Symptoms improve maximally at 3 months but may partially recur by 6 months (33), arguing for monitoring through at least 6 months.

Psychological factors are proposed to act on nutrition via two routes, neither tested against a nutritional endpoint in this population. The first is appetite, often stated as though anxiety and depression uniformly reduce intake; the wider evidence does not support that, since appetite change in depression is bidirectional and marks subgroups with opposite endocrine, metabolic and immune profiles (34). Where intake is already mechanically constrained, the net contribution of mood could be substantial, negligible, or opposite across subgroups, and no study distinguishes these. The second, more tractable route is adherence. Resilience is negatively associated with postoperative state–trait anxiety, and their interaction influences 6-month quality-of-life scores (35), implying that less resilient patients may struggle to sustain dietary and training regimens—testable by recording supplement adherence and diet-diary completeness alongside existing psychometrics, yet not so far reported. Conversely, the only direct co-observation of the two domains runs the other way, from prolonged dietary restriction to social isolation and distress (31). The most defensible reading is a bidirectional loop in which restricted eating and low mood reinforce one another, with the nutrition-to-psychology arm the better evidenced.

BDD is a distinctive concern, estimated by meta-analysis in about 14.5% of orthognathic candidates (36). Preoccupation with appearance can generate unrealistic expectations, so that even with good objective outcomes patients may remain dissatisfied with face-related functions such as eating and chewing (36). Surgery improves BDD and obsessive-compulsive symptoms, yet anxiety and depression show no significant change (37), so improvement in BDD does not equate to overall psychological recovery.

Sex differences also warrant attention. A cross-sectional analysis found higher preoperative depression and anxiety in women, who more often used engagement- and emotion-focused coping, whereas men favored disengagement coping; emotion-focused coping carried a 1.6-fold increase in depression risk (38). Because men lose more weight after surgery (3, 27), sex is a natural candidate for testing whether psychological and nutritional trajectories are linked, yet the two literatures report sex effects separately and have never been analysed together.

Clinically, psychological assessment should be routine in perioperative nutritional care: preoperative screening identifies high-anxiety, low-resilience, or BDD-positive patients needing closer monitoring, and dynamic postoperative monitoring matters equally during the three-to-six-month window of possible relapse (33). Predictors of psychiatric referral include prior psychiatric history and high-dose perioperative dexamethasone (39), whereas facial satisfaction is the strongest negative predictor (40), providing a basis for stratified intervention (Table 1). Screening is recommended here on grounds of psychological benefit and low burden; its capacity to improve nutritional outcomes remains an untested hypothesis.

## 6 Discussion: an integrated individualized framework, controversies, and research gaps

Integrating airway change, feeding function, and psychological status requires a multidimensional, dynamically adjusted framework premised on these domains interacting rather than acting independently to shape nutritional status and recovery. That premise is a hypothesis rather than a demonstrated causal structure, and Figure 1 should be read accordingly: the feeding-function arm rests on measured nutritional outcomes, whereas the airway and psychological arms are provisional mediators awaiting the studies outlined below. Stating this asymmetry identifies where the field’s evidence must be built.

From this integrative perspective, an individualized strategy comprises three elements. First, comprehensive preoperative assessment covering airway morphology (CBCT-derived pharyngeal volume and minimum cross-sectional area), masticatory indices (bite force, electromyographic activity), and validated psychological instruments (State–Trait Anxiety Inventory, BDD screening), identifying high-risk patients: those undergoing large mandibular setback, those with pre-existing masticatory dysfunction, and those with high anxiety or low resilience. Second, postoperative regimens stratified by procedure, direction of airway change, and psychological status, with early avoidance of supine feeding where airway volume is markedly reduced. Third, rehabilitation integrating masticatory training with resilience-building; current evidence supports introducing functional training around 3 months (8, 9), and mindful self-care training (41) may act synergistically. These elements differ in evidential standing: staged texture progression, protein-focused supplementation and body-composition monitoring follow from measured nutritional data, whereas airway-informed feeding positioning and psychological screening are low-cost, independently justifiable precautions that should not yet be presented as nutritionally proven.

Several controversies merit attention. First, the clinical significance of post-setback OSA risk is disputed: some studies argue that volume reduction is insufficient to cause clinical OSA in non-obese young Class III patients (42), while others report increased incidence (19, 21); heterogeneity in demographics, diagnostic criteria, and follow-up likely underlies the discrepancy, and standardized polysomnography controlling for BMI, age, and baseline airway is needed. Second, as noted above, the efficacy of masticatory training is unconfirmed (8, 9). Third, the psychological-nutritional relationship is less controversial than unexamined: associations between psychological factors and nutritional outcomes have not been established in orthognathic patients, and no study has tested whether psychological intervention improves them.

Research gaps are notable. First, interactions among airway change, masticatory recovery, and psychological status are unknown; whether airway reduction worsens depression via impaired sleep, thereby reducing appetite and intake, remains untested. Closing this gap need not require large new trials. A prospective cohort adding three inexpensive layers to routine follow-up, namely a validated intake record, serial bioimpedance, and repeated sleep and mood measures, collected at existing imaging and psychometric visits, would allow mediation analysis to convert both inferred links into measured ones. Reporting nutritional endpoints in airway and psychological studies should become the default: it is the absence of these endpoints, not of patients, that limits the field. Second, most nutritional-intervention studies are small, single-center efforts; dietary heterogeneity reflects the absence of evidence-based guidelines (4), and multicenter trials are needed to define optimal texture-progression and ONS strategies. Third, long-term follow-up is scarce: most studies extend only 3–6 months, yet weight recovery may take over 4 months and muscle mass may remain below baseline at 6 months (4, 27). Fourth, subgroups such as older, obese, or temporomandibular-disorder patients lack individualized strategies (4, 43). Methodologically, the formal appraisal (Supplementary Tables S3–S8) confirms recurrent problems: small non-randomized designs, heterogeneous airway-measurement methods (7), inconsistent psychological instruments that limit meta-analysis, and failure to control confounders such as preoperative nutritional status and analgesia.

## 7 Conclusions and future perspectives

Perioperative nutritional management in orthognathic surgery spans airway morphology, feeding function, and psychological status. Although surgery substantially improves quality of life (43, 44), early postoperative nutritional challenges are considerable, with common loss of weight and muscle mass that recovers over months. Adaptive changes in mastication and swallowing offer rehabilitation targets, but optimal training design remains undefined; psychological factors, especially preoperative anxiety and postoperative depression, are hypothesized to affect eating behavior and adherence, though not yet demonstrated against nutritional endpoints. An individualized strategy should rest on multidimensional preoperative assessment and adapt to the surgical movement, direction of airway change, and psychological profile, delivered through multidisciplinary collaboration among maxillofacial surgeons, dietitians, psychologists, and physiotherapists. Priorities for future research include multicenter randomized trials of dietary and supplementation strategies, standardized assessment tools, and risk-stratified decision aids. The most valuable near-term contribution, however, would be simpler: that airway and psychological studies in these patients begin to report what patients actually eat and what happens to their body composition. Digital tools may support real-time intake recording, weight tracking and personalized guidance, while patient-reported outcome measures can identify patients needing additional support. Ultimately, the goal is not only to maintain nutritional status and promote healing but to improve patients’ overall recovery experience and quality of life.
