# Supplementary material for: Individualized perioperative nutritional management in orthognathic surgery: an evidence-graded, integrated framework linking upper-airway change, feeding function, and psychological status
Source: Front Nutr. 2026 Sep 8;13:1944347. doi: 10.3389/fnut.2026.1944347 (PMC13595193; doi:10.3389/fnut.2026.1944347)
Supplement: Supplementary file 1 [file Table_1.docx]

**Supplementary Material**

This Supplementary Material reports, in full, the literature-search parameters and eligibility criteria summarised in section 2 of the main text, together with the item-level methodological quality appraisal of every study used to support a domain-to-nutrition link. Supplementary Table S8 brings the two together, reporting for each asserted link both its proximity to a measured nutritional endpoint and the methodological quality of the studies that support it. Reference numbers in square brackets correspond to the reference list of the main article.

**Supplementary Table S1. Database-specific search strategies**

| **Database and platform** | **Date range searched** | **Search string as run** |
| --- | --- | --- |
| PubMed / MEDLINE | Inception – 30 June 2026 | ("orthognathic surgery"[MeSH] OR orthognathic*[tiab] OR "dentofacial deformit*"[tiab] OR "sagittal split osteotom*"[tiab] OR "Le Fort I"[tiab] OR "maxillomandibular advancement"[tiab] OR "mandibular setback"[tiab] OR "bimaxillary surgery"[tiab]) AND (nutrition*[tiab] OR "nutritional status"[MeSH] OR diet*[tiab] OR "weight loss"[MeSH] OR "body composition"[MeSH] OR "energy intake"[MeSH] OR "dietary supplements"[MeSH] OR albumin[tiab] OR sarcopenia[tiab] OR "upper airway"[tiab] OR "pharyngeal airway"[tiab] OR "sleep apnea, obstructive"[MeSH] OR "apnea-hypopnea"[tiab] OR mastication[MeSH] OR masticat*[tiab] OR deglutition[MeSH] OR swallow*[tiab] OR "bite force"[MeSH] OR "tongue pressure"[tiab] OR anxiety[MeSH] OR depression[MeSH] OR "body dysmorphic disorders"[MeSH] OR resilience[tiab] OR "quality of life"[MeSH]) |
| Embase (Elsevier) | Inception – 30 June 2026 | ('orthognathic surgery'/exp OR orthognathic*:ti,ab OR 'dentofacial deformity':ti,ab OR 'sagittal split osteotomy':ti,ab OR 'Le Fort I':ti,ab OR 'maxillomandibular advancement':ti,ab OR 'mandibular setback':ti,ab) AND ('nutritional status'/exp OR 'diet therapy'/exp OR 'body weight loss'/exp OR 'body composition'/exp OR 'caloric intake'/exp OR 'nutritional support'/exp OR 'upper respiratory tract'/exp OR 'sleep disordered breathing'/exp OR 'mastication'/exp OR 'swallowing'/exp OR 'anxiety'/exp OR 'depression'/exp OR 'body dysmorphic disorder'/exp OR 'psychological resilience'/exp) |
| Web of Science Core Collection | Inception – 30 June 2026 | TS=(orthognathic* OR "dentofacial deformit*" OR "sagittal split osteotom*" OR "Le Fort I" OR "maxillomandibular advancement" OR "mandibular setback") AND TS=(nutrition* OR diet* OR "weight loss" OR "body composition" OR "energy intake" OR "oral nutritional supplement*" OR "upper airway" OR "obstructive sleep apnea" OR masticat* OR swallow* OR deglutition OR "bite force" OR "tongue pressure" OR anxiety OR depress* OR "body dysmorphic" OR resilience) |
| Cochrane Library (CENTRAL) | Inception – 30 June 2026 | (orthognathic OR "dentofacial deformity" OR "sagittal split osteotomy" OR "Le Fort I" OR "maxillomandibular advancement" OR "mandibular setback"):ti,ab,kw AND (nutrition OR diet OR "weight loss" OR "body composition" OR "oral nutritional supplement" OR "upper airway" OR "sleep apnoea" OR mastication OR swallowing OR anxiety OR depression):ti,ab,kw |
| Supplementary sources | 30 June 2026 | Backward citation screening of the reference lists of all included articles and of relevant systematic reviews; forward citation checking in Web of Science and Google Scholar for the key nutritional-outcome cohorts. Targeted searches were additionally run for the small number of studies outside orthognathic populations that are cited explicitly in the review to test the direction of an inferred link (experimental sleep restriction and energy intake; appetite phenotypes in depression). |

Searches were run by one author and independently re-run by a second author on the same date to confirm reproducibility. No date or study-design limits were applied at the search stage; restriction to English-language full text was applied at screening.

**Supplementary Table S2. Inclusion and exclusion criteria**

| **Criterion** | **Included** | **Excluded** |
| --- | --- | --- |
| Population | Adolescent or adult patients with dentofacial deformity undergoing, or scheduled to undergo, orthognathic surgery (Le Fort I, bilateral sagittal split osteotomy, bimaxillary surgery, genioplasty as an adjunct, maxillomandibular advancement or setback). | Patients with craniofacial syndromes (e.g. cleft lip and palate, Treacher Collins, Crouzon) as the sole population; facial trauma; head-and-neck oncological resection or free-flap reconstruction; distraction osteogenesis for congenital deformity; temporomandibular joint replacement alone; purely orthodontic (non-surgical) cohorts. |
| Exposure / topic | Perioperative nutritional management; surgically induced upper-airway change and sleep-disordered breathing; masticatory or swallowing function; perioperative psychological status (anxiety, depression, body dysmorphic disorder, resilience, coping). | Studies addressing only surgical technique, fixation hardware, skeletal stability, condylar position, aesthetic morphometrics, anaesthetic or analgesic protocols, or cost, with no reported nutritional, airway, feeding-function or psychological outcome. |
| Outcomes | At least one of: energy or macronutrient intake; body weight or BMI; body composition (bioimpedance, anthropometry); nutrition-related biochemistry (haemoglobin, albumin, prealbumin); pharyngeal volume or minimum cross-sectional area; apnoea–hypopnoea index or validated sleep measures; bite force, electromyographic activity, mouth opening, tongue pressure, swallowing kinematics; validated psychometric instruments; oral-health- or oral-function-related quality of life. | Studies reporting only surgeon-rated aesthetic outcome, cephalometric hard-tissue measurement without airway data, or complication rates without any of the listed outcomes. |
| Study design | Systematic reviews and meta-analyses; randomised controlled trials; non-randomised intervention studies; prospective and retrospective cohort studies; cross-sectional and case–control studies; qualitative studies. Narrative reviews were retained only as background or to frame a stated controversy, and were not used as primary evidence for any domain-to-nutrition link. | Case reports and case series with fewer than 10 participants; conference abstracts, letters and editorials without full-text data; animal, cadaveric and purely in-silico or finite-element studies; protocols without results; duplicate reports of the same cohort (the most complete report was retained). |
| Language and date | English-language full text; databases searched from inception to 30 June 2026 with no lower date restriction, to allow the historical trajectory of each domain to be represented. | Non-English full text where no validated English version was available. |
| Studies outside the orthognathic population | Eligible only where cited explicitly and transparently to test the plausibility or direction of an inferred link (experimental sleep restriction and energy intake; appetite phenotypes in depression). Such studies are labelled as external evidence throughout and are never counted as direct evidence for an orthognathic link. | General-population evidence introduced to substitute for missing orthognathic data, or used to assert a direction of effect in orthognathic patients. |

**Study selection and data handling**

Records retrieved from the four databases were de-duplicated and screened on title and abstract by two authors independently; full texts of all potentially eligible records were then assessed against the criteria in Supplementary Table S2. Disagreement at either stage was resolved by discussion, with the third author adjudicating where consensus was not reached. Because this is a narrative mini-review whose purpose is integrative synthesis rather than exhaustive enumeration or quantitative pooling, no PRISMA flow count is reported and no attempt was made to identify every eligible record; where several reports addressed the same question, priority was given to systematic reviews, meta-analyses and randomised trials, and thereafter to prospective studies reporting a measured nutritional endpoint. This selective, purposive approach is a recognised limitation of the narrative format and is stated here so that readers can judge the strength of the underlying evidence base for themselves.

**Methodological quality appraisal: approach**

Every study cited in support of a domain-to-nutrition link was appraised with the instrument appropriate to its design: the Newcastle–Ottawa Scale (NOS) for cohort studies and, in its adapted form, for cross-sectional studies; the Cochrane RoB 2 tool for randomised trials; ROBINS-I for non-randomised studies of interventions; AMSTAR 2 for systematic reviews and meta-analyses; and the Critical Appraisal Skills Programme (CASP) qualitative checklist for qualitative studies. Two authors appraised each study independently and disagreements were resolved by discussion. Narrative reviews cited only as background or to frame a stated controversy were not formally appraised and were not used as primary evidence for any link; these are references [1] and [42].

Two conventions should be noted. First, most of the surgical cohorts are single-arm pre–post designs with no unexposed comparison group, so NOS item S2 (selection of the non-exposed cohort) cannot be satisfied; the maximum attainable score for these studies is therefore 8 of 9 rather than 9 of 9, and this is a property of the design rather than a defect in conduct. Second, methodological quality and evidence proximity are independent axes. A methodologically strong study that measures pharyngeal volume but not dietary intake still yields only indirect evidence for a nutritional link, and a modest cohort that measures intake directly may support a nutritional inference more strongly. Supplementary Table S8 therefore reports both dimensions side by side, and it is their conjunction, not either alone, that determines the confidence expressed in the main text.

**Supplementary Table S3. Newcastle–Ottawa Scale: cohort studies**

| **Study (reference in main text)** | **Design** | **S1** | **S2** | **S3** | **S4** | **C** | **O1** | **O2** | **O3** | **Total** | **Quality** |
| --- | --- | --- | --- | --- | --- | --- | --- | --- | --- | --- | --- |
| Inaba et al., 2023 [2] | Prospective single-arm cohort | 1 | 0 | 1 | 1 | 1 | 1 | 1 | 1 | 7/9 | Good |
| Mogensen et al., 2025 [3] | Prospective descriptive cohort | 1 | 0 | 1 | 1 | 2 | 1 | 1 | 1 | 8/9 | Good |
| Giralt-Hernando et al., 2021 [18] | Consecutive cohort, 3D imaging | 1 | 0 | 1 | 1 | 1 | 1 | 1 | 1 | 7/9 | Good |
| Kongsong et al., 2025 [19] | Prospective cohort with polysomnography | 1 | 0 | 1 | 1 | 1 | 1 | 1 | 1 | 7/9 | Good |
| Genc et al., 2020 [24] | Prospective cohort vs healthy volunteers | 1 | 1 | 1 | 1 | 1 | 1 | 1 | 1 | 8/9 | Good |
| Celik et al., 2024 [26] | Prospective longitudinal cohort | 1 | 0 | 1 | 1 | 1 | 1 | 1 | 1 | 7/9 | Good |
| Gasimov et al., 2025 [27] | Prospective cohort, bioimpedance | 1 | 0 | 1 | 1 | 1 | 1 | 1 | 1 | 7/9 | Good |
| Bayram et al., 2024 [30] | Prospective cohort, bimaxillary only | 1 | 0 | 1 | 1 | 1 | 1 | 1 | 0 | 6/9 | Fair |
| Yang et al., 2026 [35] | Prospective cohort, 6-month follow-up | 1 | 0 | 1 | 1 | 2 | 1 | 1 | 1 | 8/9 | Good |
| Haberle et al., 2020 [37] | Prospective pre–post cohort | 1 | 0 | 1 | 1 | 1 | 1 | 1 | 0 | 6/9 | Fair |
| Kettunen et al., 2023 [39] | Retrospective record-based cohort | 1 | 0 | 1 | 1 | 1 | 1 | 1 | 1 | 7/9 | Good |
| Kao et al., 2025 [40] | Observational pre–post cohort | 1 | 0 | 1 | 1 | 1 | 1 | 1 | 0 | 6/9 | Fair |

S1, representativeness of the exposed cohort; S2, selection of the non-exposed cohort; S3, ascertainment of exposure; S4, demonstration that the outcome of interest was not present at the start of the study; C, comparability of cohorts based on design or analysis (maximum 2); O1, assessment of outcome; O2, follow-up long enough for outcomes to occur; O3, adequacy of follow-up. Ratings: good, 7–9; fair, 4–6; poor, 0–3.

**Supplementary Table S4. Newcastle–Ottawa Scale (adapted): cross-sectional studies**

| **Study (reference in main text)** | **Design** | **S1** | **S2** | **S3** | **S4** | **C** | **O1** | **O2** | **Total** | **Quality** |
| --- | --- | --- | --- | --- | --- | --- | --- | --- | --- | --- |
| Zhou et al., 2024 [10] | Cross-sectional, early postoperative | 1 | 0 | 0 | 2 | 2 | 2 | 1 | 8/10 | Good |
| Kok et al., 2023 [11] | Multicentre cross-sectional | 1 | 1 | 1 | 2 | 1 | 2 | 1 | 9/10 | Good |
| Zeng et al., 2026 [12] | Cross-sectional, preoperative | 1 | 0 | 0 | 2 | 1 | 2 | 1 | 7/10 | Good |
| Huang et al., 2021 [22] | Cross-sectional, sleep and satisfaction | 1 | 0 | 0 | 2 | 1 | 2 | 1 | 7/10 | Good |
| Nagasaki et al., 2024 [28] | Comparative cross-sectional, instrumented | 0 | 0 | 1 | 2 | 1 | 2 | 1 | 7/10 | Good |
| Avramut et al., 2023 [38] | Cross-sectional, sex-stratified | 1 | 0 | 0 | 2 | 1 | 2 | 1 | 7/10 | Good |
| Simmons et al., 2020 [34] — external evidence | Cross-sectional, non-orthognathic | 0 | 0 | 0 | 2 | 2 | 2 | 1 | 7/10 | Good |

S1, representativeness of the sample; S2, justification of sample size; S3, characterisation of non-respondents; S4, ascertainment of the exposure or risk factor (maximum 2); C, comparability of groups by design or analysis (maximum 2); O1, assessment of the outcome (maximum 2); O2, appropriateness of the statistical test. Ratings: good, 7–10; fair, 4–6; poor, 0–3.

**Supplementary Table S5. RoB 2 and ROBINS-I: randomised and non-randomised intervention studies**

| **Study (reference in main text)** | **Design** | **Tool** | **Domain-level judgements** | **Overall judgement** |
| --- | --- | --- | --- | --- |
| Yang et al., 2020 [9] | Single-centre randomised trial (stated as preliminary) | RoB 2 | Randomisation process: some concerns (allocation concealment not fully described). Deviations from intended interventions: some concerns (participants and therapists not blinded to a behavioural intervention). Missing outcome data: low. Measurement of the outcome: some concerns (outcome assessment not reported as blinded). Selection of the reported result: some concerns (no pre-registered analysis plan identified). | Some concerns |
| Prachasartta et al., 2024 [29] | Randomised clinical trial of oral nutritional supplementation | RoB 2 | Randomisation process: low. Deviations from intended interventions: some concerns (open-label supplement; adherence not independently verified). Missing outcome data: low. Measurement of the outcome: low for anthropometry, some concerns for self-reported oral-health-related quality of life. Selection of the reported result: low. | Some concerns |
| Ghanipour et al., 2025 [41] | Trial of mindful self-care training | RoB 2 | Randomisation process: some concerns. Deviations from intended interventions: high (unblinded psychological intervention). Missing outcome data: some concerns. Measurement of the outcome: high (self-reported body-image and self-esteem outcomes with unblinded participants). Selection of the reported result: some concerns. | High |
| Kawai et al., 2020 [8] | Non-randomised study of functional (chewing-gum) training | ROBINS-I | Confounding: serious — spontaneous postoperative recovery of masticatory function cannot be separated from the training effect in the absence of a concurrent untrained control. Selection of participants: moderate. Classification of interventions: low. Deviations from intended interventions: moderate. Missing data: low. Measurement of outcomes: moderate (electromyography is objective, but assessors were not reported as blinded). Selection of the reported result: moderate. | Serious risk of bias |

**Supplementary Table S6. AMSTAR 2: systematic reviews and meta-analyses**

| **Review (reference in main text)** | **Topic** | **Principal limitations against AMSTAR 2 critical domains** | **Overall confidence** |
| --- | --- | --- | --- |
| Irgebay et al., 2023 [4] | Diet protocols and postoperative weight loss | No a priori protocol registered (item 2); search strategy incompletely reported (item 4); no formal risk-of-bias assessment of included studies (item 9); heterogeneity of dietary schemes not accounted for in the synthesis (item 13). | Critically low |
| AlQahtani et al., 2023 [5] | Nutritional state after orthognathic surgery | Protocol not registered (item 2); list of excluded studies not provided (item 7); publication bias not investigated (item 15). | Low |
| Shokri et al., 2021 [6] | Upper-airway change by procedure (CBCT) | Protocol not registered (item 2); list of excluded studies not provided (item 7); heterogeneity of airway-segmentation methods discussed but not modelled (item 13). | Low |
| Steegman et al., 2023 [7] | CBCT volumetric airway change | List of excluded studies not provided (item 7); publication bias not formally assessed (item 15). Risk of bias of primary studies was assessed and used in interpretation. | Moderate |
| Grinberg et al., 2025 [20] | Posterior airway compromise in class III | Protocol registration unclear (item 2); list of excluded studies not provided (item 7); limited investigation of publication bias (item 15). | Low |
| Wei et al., 2024 [21] | Factors influencing post-setback OSA | Protocol not registered (item 2); list of excluded studies not provided (item 7); no meta-analytic handling of clinical heterogeneity (item 13). | Low |
| Daluz et al., 2022 [25] | Long-term airway evolution | No a priori protocol (item 2); search restricted and incompletely reported (item 4); risk of bias of included studies not assessed (item 9); publication bias not assessed (item 15). | Critically low |
| Lin et al., 2026 [33] | Depression and anxiety trajectories | List of excluded studies not provided (item 7); otherwise protocol-driven, with risk-of-bias assessment incorporated into the synthesis. | Moderate |
| Saenz-Ravello et al., 2025 [36] | Prevalence of body dysmorphic disorder | List of excluded studies not provided (item 7); prevalence heterogeneity attributable to differing screening instruments only partly explained (item 13). | Moderate |
| Zheng et al., 2025 [43] | Oral-function-related quality of life | Protocol registration unclear (item 2); list of excluded studies not provided (item 7); publication bias not assessed (item 15). | Low |
| Meger et al., 2021 [44] | Quality of life after orthognathic surgery | List of excluded studies not provided (item 7); otherwise registered protocol with risk-of-bias assessment and adequate handling of heterogeneity. | Moderate |
| Zhu et al., 2019 [23] — external evidence | Sleep restriction and metabolic parameters (RCTs, healthy adults) | Registered protocol, comprehensive search, risk of bias assessed and incorporated, heterogeneity and publication bias formally investigated. Cited only as external evidence on the direction of the sleep–intake relationship. | High |

AMSTAR 2 critical domains are items 2 (protocol registered before commencement), 4 (adequacy of the literature search), 7 (justification for excluding individual studies), 9 (satisfactory technique for assessing risk of bias), 11 (appropriateness of meta-analytical methods), 13 (accounting for risk of bias when interpreting results) and 15 (assessment of publication bias). Overall confidence: high, no or one non-critical weakness; moderate, more than one non-critical weakness; low, one critical flaw; critically low, more than one critical flaw.

**Supplementary Table S7. CASP checklist: qualitative studies**

| **Study (reference in main text)** | **CASP items met** | **Items rated unclear or not met** | **Overall assessment** |
| --- | --- | --- | --- |
| Hansen et al., 2026 [31] | 9 of 10 | The relationship between researcher and participants (reflexivity) is only briefly addressed. Aims, qualitative methodology, design, recruitment, data collection, ethics, analytic rigour, statement of findings and value of the research were all judged adequate; findings are directly relevant to the nutrition-to-psychology direction of effect. | High confidence; include |
| Kok et al., 2025 [32] | 10 of 10 | No item judged inadequate. Purposive sampling, data saturation, analytic framework and reflexivity are explicitly reported; findings on the multifactorial nature of preoperative anxiety are well supported by the presented data. | High confidence; include |

**Supplementary Table S8. Link-level synthesis: evidence proximity and methodological quality for each domain-to-nutrition link**

| **Link asserted in the review** | **Evidence proximity** | **Supporting studies (design)** | **Methodological quality of that evidence** | **Overall confidence in the link** |
| --- | --- | --- | --- | --- |
| Mandibular setback reduces, and advancement enlarges, upper-airway volume and minimum cross-sectional area | Indirect | [6], [7], [20], [25] (systematic reviews/meta-analyses); [18], [19] (cohorts) | AMSTAR 2: one moderate, two low, one critically low. NOS: 7/9 and 7/9. Direction of effect consistent across reviews of differing quality. | Moderate for the airway change itself; the nutritional consequence is not evidenced |
| Reduced airway volume after setback increases the risk of obstructive sleep apnoea | Indirect | [19] (prospective cohort with polysomnography); [20], [21] (systematic reviews); [42] (narrative review, contrary position, not appraised) | NOS 7/9; AMSTAR 2 low for both reviews. Incidence estimates vary with procedure, setback magnitude and diagnostic criteria. | Low; actively disputed in the literature |
| Sleep-disordered breathing reduces energy intake or impairs nutritional recovery | Inferred | No orthognathic study. [22] (cross-sectional; related sleep to satisfaction and quality of life, not to intake or weight). [23] (external meta-analysis of randomised sleep-restriction trials, showing increased intake) | NOS 7/10 for [22]; AMSTAR 2 high for [23], but in a non-orthognathic population and pointing in the opposite direction. | Very low; direction of effect unresolved |
| Airway and hyoid repositioning alters swallowing mechanics | Indirect | [24] (prospective electromyographic cohort with healthy comparison group) | NOS 8/9. Swallowing physiology measured; bolus volume, meal duration and energy delivered were not. | Moderate for the physiological change; very low for any nutritional consequence |
| Impaired mastication reduces energy and macronutrient intake | Direct | [26] (prospective longitudinal cohort with dietary records); [28] (comparative cross-sectional, tongue pressure); [8] (non-randomised training study) | NOS 7/9 and 7/10; ROBINS-I serious for [8]. Intake measured against the timing of functional recovery in a single-centre cohort. | Moderate |
| Postoperative dietary restriction causes weight and BMI loss | Direct | [2], [3], [27] (prospective cohorts); [4], [5] (systematic reviews) | NOS 7/9, 8/9, 7/9. AMSTAR 2: one low, one critically low. Cohort evidence is consistent; the pooled magnitude rests on low-confidence reviews. | Moderate to high for the direction; low for the pooled magnitude |
| Dietary restriction depletes lean mass and alters nutrition-related biochemistry | Direct | [27] (bioimpedance cohort); [2], [30] (cohorts with serial haemoglobin and albumin) | NOS 7/9, 7/9, 6/9. All single-centre, modest samples; bioimpedance in the presence of early postoperative oedema is a recognised source of measurement error. | Low to moderate |
| Oral nutritional supplementation attenuates postoperative weight loss | Direct | [29] (randomised clinical trial) | RoB 2: some concerns (open-label supplementation). Single trial; the between-group difference at weeks 2 and 4 was not maintained at week 12. | Low |
| Masticatory training accelerates functional recovery | Direct (functional endpoint) | [8] (non-randomised training study); [9] (randomised trial of a chewing exerciser) | ROBINS-I serious (spontaneous recovery inseparable from the training effect); RoB 2 some concerns. The two studies disagree. | Very low; evidence conflicting |
| Preoperative anxiety and body dysmorphic disorder are prevalent in this population | Contextual (not a nutritional link) | [11], [12], [38] (cross-sectional); [36] (meta-analysis of prevalence); [32] (qualitative) | NOS 9/10, 7/10, 7/10; AMSTAR 2 moderate; CASP 10/10. Prevalence estimates vary with the screening instrument used. | Moderate |
| Psychological status modifies appetite and therefore intake | Inferred | No orthognathic study measures a nutritional endpoint as a function of psychological status. [34] (external cross-sectional evidence that appetite change in depression is bidirectional) | NOS 7/10 for [34], in a non-orthognathic population. No orthognathic evidence of any quality exists for this link. | Very low; direction of effect unresolved |
| Psychological status affects adherence and hence nutritional recovery | Inferred | [35] (prospective cohort relating resilience and anxiety to 6-month quality of life); [10], [33] (symptom trajectories) | NOS 8/9 and 8/10; AMSTAR 2 moderate. Adherence to dietary or rehabilitation regimens has never been recorded in this population. | Very low; testable with existing cohorts |
| Prolonged dietary restriction causes social isolation and psychological distress (reverse direction) | Direct co-observation | [31] (qualitative study of patient experience) | CASP 9/10, high confidence. Single qualitative study; the direction is well evidenced but the magnitude is not quantified. | Low to moderate; the better-evidenced arm of the psychological loop |
| Airway, feeding function and psychological status interact to determine nutritional trajectory | Untested | No study measures cross-domain mediation. | Not assessable. No study collects airway, feeding, psychological and nutritional variables concurrently in the same cohort. | Not assessable; hypothesis-generating only |

Evidence proximity: direct, a nutritional endpoint (intake, weight, body composition or nutrition-related biochemistry) was measured in orthognathic patients as a function of the domain variable; indirect, only an intermediate endpoint was measured; inferred, the link rests on mechanism or on extrapolation from other populations; untested, no study addresses the link. Overall confidence reflects the conjunction of proximity and methodological quality, and is the basis for the graded claims made in the main text and in Table 1.

**Supplementary References**

S1. Wells GA, Shea B, O’Connell D, Peterson J, Welch V, Losos M, et al. The Newcastle-Ottawa Scale (NOS) for assessing the quality of nonrandomised studies in meta-analyses. Ottawa: Ottawa Hospital Research Institute; 2021.

S2. Modesti PA, Reboldi G, Cappuccio FP, Agyemang C, Remuzzi G, Rapi S, et al. Panethnic differences in blood pressure in Europe: a systematic review and meta-analysis. PLoS One. 2016;11(1):e0147601. [source of the adapted Newcastle-Ottawa Scale for cross-sectional studies]

S3. Sterne JAC, Savović J, Page MJ, Elbers RG, Blencowe NS, Boutron I, et al. RoB 2: a revised tool for assessing risk of bias in randomised trials. BMJ. 2019;366:l4898.

S4. Sterne JA, Hernán MA, Reeves BC, Savović J, Berkman ND, Viswanathan M, et al. ROBINS-I: a tool for assessing risk of bias in non-randomised studies of interventions. BMJ. 2016;355:i4919.

S5. Shea BJ, Reeves BC, Wells G, Thuku M, Hamel C, Moran J, et al. AMSTAR 2: a critical appraisal tool for systematic reviews that include randomised or non-randomised studies of healthcare interventions, or both. BMJ. 2017;358:j4008.

S6. Critical Appraisal Skills Programme. CASP Qualitative Studies Checklist. Oxford: CASP UK; 2018.
